# Diseases of the Lungs and Pleura

**Published:** 1887-02

**Authors:** 


Diseases of the Lungs and Pleuræ, Including Consumption.—By R. Douglas Powell, M. D., Lond., Fellow of the Royal College of Physicians; Physician to the Middlesex Hospital and to the Hospital for Consumption and Diseases of the Chest, at

Brompton; late Assistant Physician and Lecturer on Materia Medica at the Charing Cross Hospital. Third edition, rewritten and enlarged, with illustrations, including two lithographic plates; being Vol. XI. of Wood's Library for 1886 (12 vols. in set, price, $15.00). New York, William Wood & Company.

